# Supplementary material for: Rapid turnover of life-cycle-related genes in the brown algae
Source: Genome Biol. 2019 Feb 14;20:35. doi: 10.1186/s13059-019-1630-6 (PMC6374913; doi:10.1186/s13059-019-1630-6)
Supplement: Supplementary file 3 — Protein alignments, corresponding Gblocks html file and CDS sequences for the lineage conserved gametophyte-biased genes. (ZIP 2109 kb) [file 13059_2019_1630_MOESM3_ESM.zip › Con_GA_alignments/Ec-12_006240.aa_ali.fasta-gb.htm]

Ec-12\_006240.aa.fasta.trans.aa\_ali.fasta


## Gblocks 0.91b Results

Processed file: **Ec-12\_006240.aa.fasta.trans.aa\_ali.fasta**  
Number of sequences: **2**  
Alignment assumed to be: **Protein**  
New number of positions: **260** (selected positions are underlined in blue)

```
                         10        20        30        40        50        60
                 =========+=========+=========+=========+=========+=========+
Ec-12_006240     ------------------------------------------------------------
MSTRG.23717.1    PYWFARVDQSSCSSCSSCSSSMFSFLSALTVLMWPSRHVLVFGYDVITKVDSMLEMLQGK
                                                                             


                         70        80        90       100       110       120
                 =========+=========+=========+=========+=========+=========+
Ec-12_006240     ------------------------------------------------------------
MSTRG.23717.1    TAWHSTIKERDLREALDNCVAAVKSDALNDGHTKRLLGLLRLVIDDRQRFVLFEDVEQAL
                                                                             


                        130       140       150       160       170       180
                 =========+=========+=========+=========+=========+=========+
Ec-12_006240     ------------------------------------------------------------
MSTRG.23717.1    LEPRGNTQTREVAGLLGANLAQAMDRPGRHAADPSVQEAFLRHSIRTFPENPVASRSLGY
                                                                             


                        190       200       210       220       230       240
                 =========+=========+=========+=========+=========+=========+
Ec-12_006240     ----------------------------------------------MFRFSRVSAETRRL
MSTRG.23717.1    KLEQSGQEQRAMELYFEGLVRDPERLDLRLLLASSCSPFLNDTSEGIMRYARISGGIGRL
                                                                 ############


                        250       260       270       280       290       300
                 =========+=========+=========+=========+=========+=========+
Ec-12_006240     VEHIQTSDAPLRNVPNPGRDLIHIPSFSWPYLGRNMRPLMESLCWILAAMTVGLHEAIAR
MSTRG.23717.1    RHYIQTSAHPLPNVASPGRDLAYIPSYSWAYLGYSMRFLMESMCWILAAITAGLHEAAGG
                 ############################################################


                        310       320       330       340       350       360
                 =========+=========+=========+=========+=========+=========+
Ec-12_006240     LQPRNAWDNSTSSGRQGTRGAFGEGEQPQISLTPPPSIATKDVRITVGFVMERWGNRSPH
MSTRG.23717.1    LQPQAGWASEDQEGLETEKGPFEGEGPPQRFANRRLPPTARHRRIVLGVVMERHGNQSPH
                 #############################              #################


                        370       380       390       400       410       420
                 =========+=========+=========+=========+=========+=========+
Ec-12_006240     RLIQGVLEGMDRSRFRLVVFSQDYTGYYCEAGKALLEAAEEVKIFPWHPFAEGLTDPFAD
MSTRG.23717.1    RLIQGVLEGLDRAKFRLVMFTRDYVAEYSDAGQALLKLADEVLVLSWHPFAKGLADPFAD
                 ############################################################


                        430       440       450       460       470       480
                 =========+=========+=========+=========+=========+=========+
Ec-12_006240     RGVIA---------------------------SVQVVFGHGHPVTSGSPGIDYFVSSDLF
MSTRG.23717.1    RGVVSAAQVDVLLYTALGNTLWSHLLALGRLAPVQIAFGHGHPVTSGSPGIDYFVSSDLF
                 ###                              ###########################


                        490       500       510       520       530       540
                 =========+=========+=========+=========+=========+=========+
Ec-12_006240     ETTASIDARELRRIAGTPSDLSLATTAVTP------------------------------
MSTRG.23717.1    EKRISIDAREALRVEDAESDVSPAATTADANVYFGEAAGTIVSEYGQSYPHRKLPQTKAH
                 ##########################                                  


                        550       560       570       580       590       600
                 =========+=========+=========+=========+=========+=========+
Ec-12_006240     HAGAEGCDDDD--SVHSPTAGG---------TCRADPLLSDDDQPHHYQNLPRNTTHQTL
MSTRG.23717.1    HVPAEEKSDSSSTSTEQPVLGGDGPRDYAEQFVLFDSLTASLPEPHGPVNAPSSAVEATL
                                                    #########################


                        610       620       630       640       650       660
                 =========+=========+=========+=========+=========+=========+
Ec-12_006240     AEEK-----------------------Q--------------------------------
MSTRG.23717.1    AAVRSSTPGAKVSLLEGDHLYHCIQHSKKFHPDFDVVLRGVLQSDPAAKILLAAGSEVHF
                 #                                                           


                        670       680       690       700       710       720
                 =========+=========+=========+=========+=========+=========+
Ec-12_006240     -----NL-----------------------------------------------------
MSTRG.23717.1    SRWERTLGPDLTQRLVFLPFLAYPEMLIVVASCRVMLDTFPWGAGVTSMEALLSGVPVVT
                                                                             


                        730       740       750       760
                 =========+=========+=========+=========+===
Ec-12_006240     -----------------------------------------EP
MSTRG.23717.1    LPARISVLPLAHGQLVELGVEDVLVATTPADLVKKAVKIASDS
```

```
Parameters used
Minimum Number Of Sequences For A Conserved Position: 2
Minimum Number Of Sequences For A Flanking Position: 2
Maximum Number Of Contiguous Nonconserved Positions: 8
Minimum Length Of A Block: 10
Allowed Gap Positions: None
Use Similarity Matrices: Yes
```

```
Flank positions of the 4 selected block(s)
Flanks: [229  329]  [344  423]  [454  506]  [576  601]  

New number of positions in Ec-12_006240.aa.fasta.trans.aa_ali.fasta-gb:  260  (34% of the original 763 positions)
```
